# 

**Published:** 1867-05

**Authors:** 


### THE AMERICAN JOURNAL OF DENTAL SCIENCE,

EDITED BY

A. SNOWDEN PIGGOT, M.D.,

AND

F. J. S. GORGAS, M.D., D.D.S.

NATIONAL LIBRARY

JUN 18 1980

OF MEDICINE

VOL. 1.—THIRD SERIES.

LANE MEDICAL LIBRARY

17939

SAN FRANCISCO

BALTIMORE:
SNOWDEN & COWMAN.
LONDON:
TRUBNER & CO.

1867.

W1
AM45m
V.1
1867

NLM LIBRARY

### INDEX TO VOL. I.—THIRD SERIES.

A Case of Anaplasty of the Palate. E. Franklin Coates, M.D. 305
Absorption of Gases by Solids........................ 521
Absorption of Medicines........................ 313
Absorption of Medicines by the Skin........................ 424
Address to Graduating Class, Prof. Henry R. Noel, M.D........ 585
A Dislodged Tooth Replaced. H. H. Keech, D.D.S........... 452
Aim, Power and Instruments of Dental Education. Professor Austen........................ 53. 283
Air Treatment........................ 153
Alcohol on Men and Animals, Effects of........................ 631
Allen, John, D.D.S., M.D., Physical History, &c.............. 342. 385
Aluminum. Jas. B. Bean, D.D.S........................ 31
Aluminum. Its Properties and Uses........................ 422
American Academy of Dental Science........................ 474
American Dental Association........................ 138. 251
A Move in the Right Direction........................ 475
A Mistake........................ 476
A Monster. C. G. Simmons........................ 352
An Interesting Relic........................ 104
Anæsthesia. J. E. Petrequin........................ 463
Anatomy of the Cranium and Face. Claude Baxley M.D.... 529
A New Dentifrice........................ 365
A New Micrometer........................ 473
A New Styptic........................ 575
A Remarkable Invention........................ 366
A Word of Explanation........................ 52
Animal Electricity........................ 217. 519
Animal Grafts........................ 362
Answers to Querists.—Destruction of Nerves of Temporary Teeth........................ 135
Best Material for Filling Temporary Teeth............ 137
Lancing Gums for the Extraction of Temporary Teeth. 138
Methods for obtaining Partial Impressions of the Mouth........................ 196
To Prevent Rubber from Pressing into the Joints of Teeth........................ 197
Nitrous Oxide Gas. Properties Generation and Administration of........................ 249. 301. 355. 398
Apology........................ 320
Artificial Palates. A New System of, Dr. Wilhelm Suersen. 373

Assimilation of Gelatin.................................... 632
Association of the College of Dentistry.................... 35. 40
Atropine. Its harmless Effects upon Rabbits.............. 259
Austen. Prof. P. H. Dental Education.................... 53. 283
Dental Progress............1. 105. 337. 477. 552
Baltimore College of Dental Surgery...................... 579. 620
Beeswax. James F. Babcock................................ 565
Bibliographical.—The Anterior Splint. Prof. N. R. Smith M.D. 98
A Treatise on Odontalgia. S. Parsons Shaw........ 634
Chemical Essays in Reference to Dental Surgery. Prof. George Watt, M.D.,D.D.S........................ 474
Harris' Medical and Dental Dictionary. Revised by F. J. S. Gorgas, M.D.,D.D.S................ 47. 317. 368
Humboldt Medical Archives.......................... 423
Medical Gazette..................................... 368
Nature and Treatment of Decay of the teeth. Robert Arthur, M.D.,D.D.S.......................... 47
The Chemical News.................................. 579
The Dental office and Laboratory.................... 634
The New Orleans Journal of Medicine................ 579
The New Eclectic..................................... 525
The Tree of Life or Human Degeneracy. Isaac Jennings, M.D............................................ 367
The Teeth: Their Health, Disease and Treatment. J. P. H. Brown........................................ 474
The Transactions of Am. Dental Association......... 47. 474
Physicians' Hand Book. Wm. Elmer, M.D............ 423
Bichloride of Methylene.................................... 499
Bishop. H. F. D.D.S. Rise, Progress, &c., of Dentistry...... 347. 379
Bleaching Discolored Teeth. Prof. J. H. McQuillen, M.D., D.D.S.................................................. 85
Blood Corpuscles............................................ 44
Brookins. A. B. D.D.S. Fillings, &c...................... 30
Root Filling........................ 168. 274
Browne, Prof. Rufus K, M.D. Action of Red Blood Corpuscles. 431. 488
Respiratory Changes............ 330
The Red Blood Corpuscles. Notes of observation on..... 59
The Blood: its Progress and Function................ 240
Burton, W. Leigh. Dental Surgery in the late Confed. Armies. 180
Dental Notes of the late Civil War....... 440
Fit of Vulcanite Plates.................. 392
Cafeine an Antidote to Opium.............................. 517
Cancer. Remedy for,........................................ 583
Carbolate of Iodine. J. D'Oyley Evans, D.D.S.............. 393
Carbolate of Glycerine. Geo. W. Lawrence, M.D........... 628

Carbolic Acid in Surgery. George Derby, M.D.............. 403
Caries and Necrosis of Bone. Prof. Henry R. Noel, M.D..... 425. 482
Catching Cold............................................ 95
Causation of Phthisis.................................... 582
Change of Programme...................................... 528
Charcoal................................................. 152
Chemistry of the Secretions.............................. 309
Cholera and Crocodiles................................... 575
Clinical Remarks on Schmidt's Disease of Lower Jaw....... 189
Code of Dental Ethics.................................... 317
Cohesive Shred Gold. Lamms'.............................. 50
Colburn's New Base for Artificial Teeth.................. 350
Compound Comminuted Fracture of Maxillary Bones, J. M. Snyder, M.D.......................................... 89
Compression for the Cure of Inflammation................. 521
Compression of Carotids for Convulsions.................. 577
Consanguineous Marriages................................. 319
Convention of Professors of Medical Colleges............. 101
Correction............................................... 156
Correspondence.—Letters from Paris........... 80. 247. 299. 395. 618
Letter from P. H. A...................................... 82
Cryptopia................................................ 418
Cure of Double Hare-lip by a New Method. A. Hammer, M. D. 453
Cutler, Andrew S., D.D.S. Saliva......................... 537
Cyst in the Lower Jaw.................................... 260
Death from Cardiac Neuralgia. Prof. Edward Warren, M.D.. 21
Death from Chlorodyne.................................... 633
Death from the Inhalation of Ether....................... 303
Death from Swallowing Chloroform......................... 420
Decrease of Fecundity in Europe and America.............. 190
Dental College Commencements.............................
Baltimore College of Dental Surgery... 40. 620
Missouri Dental College.................. 623
Ohio College of Dental Surgery........... 41
Penn. College of Dental Surgery.......... 41
Philadelphia Dental College.............. 42
New York College of Dentistry............ 43
Dental Education......................................... 155
Dental Medicine.......................................... 320
Dental Notes on the Late Civil War. W. Leigh Burton....... 440
Dental Surgery in the Late Confederate Armies. W. Burton. 180
Dentistry as a "Fine Art." Prof. N. W. Kingsley. 72. 123. 157. 230. 290
Dentistry at the Paris Exposition........................ 623
Deodorization of Caoutchouc.............................. 261
Deodorizing India Rubber................................. 140
Differences between Kreosote and Carbolic Acid. S. C. Benbow..................................................... 613

Disease Produced by Sleeping Together.................. 522
Diseased Meat.............................................. 149
Dissections of the Dead. Prof. Gibbons, M.D................ 457
Dodge, Prof. J. Smith, M.D.,D.DS. Treatment of Pulpless
Teeth...................................................... 111
Dr. Mary Walker............................................ 201
Elixir of Vitriol and Tannin as a Hæmostatic............... 581
Errata..................................................... 156. 424
Essential Nature of Caries. Prof. Henry R. Noel, M.D. 209. 265. 321
Ether Spray in Strangulated Hernia......................... 155
Ether versus Chloroform.................................... 199. 321
Evans, J. D'Oyley, D.D.S. Letters from Paris....... 80. 247. 299. 393
Carbolate of Iodine............. 393
Magnesian Cement............. 618
Excretion of Urea.......................................... 364
Extraordinary Obesity...................................... 420
Facts and Philosophy of Dental Progress. Professor Austen
M.D.,D.D.S............................. 1. 105. 337. 477. 552
Fillings, &c. A. B. Brookins, D.D.S........................ 30
Fillings of Different metals in Contact. R. P. Nevill, D.D.S. 222
Food for Babies............................................ 467
Formation of Cells in Animal Bodies........................ 152
Fracture of Lower Jaw. New Mode of Treating................ 509
French Malleable Horn...................................... 264
Gases in Meteoric Iron..................................... 204
Gold Foil.................................................. 7
Glycerine. Testing of...................................... 473
Gunning, Thomas B., D.D.S. M.D. On the Physiological
Action of Muscles concerned in the Movements of the
Lower Jaw.................................................. 597
Gun Shot wound of the Heart. V. S. Lindsley, M.D........... 405
Harelip. Successful operation on, A. B. Stout, M.D......... 401
Hawe's Tongue and Duct Compressor.......................... 156
Heart's Action after Death................................. 472
Heat as a Resuscitating Agent.............................. 419
Heat produced by Mental Work............................... 203
Hydrophobia................................................ 206
Hoff and the New York Academy of Medicine.................. 366
Human Bite Poisonous....................................... 261
Improved Saliva Pump....................................... 635
Human Voice................................................ 578
Influence of Alcohol on Temperature of Body................ 154
Ingenious Contrivance...................................... 46
Interesting to Graduates of Balto. College of Dental Surg.. 370
Interesting to Dentists.................................... 525
Iodine Preparations for Dental Use......................... 526
Iodized Rubber............................................. 580

Iowa State Dental Society.................................... 104
Journalism.................................................. 99
Keech, H. H., D.D.S. A Dislodged Tooth Replaced....... 452
Kingsley, Prof. Norman W. Dentistry as a "Fine Art.".. 72. 123. 157
................................................. 230. 590
Leibig's Extract of Flesh.................................. 92
Local Anæsthesia by Cold.................................. 262
Magnesian Cement. J. D'Oyley Evans, D.D.S.............. 618
Magnetic Somnambulism. W. Mason Turner, M.D......... 358
Making Light of the Dead.................................. 263
Malarial Neuralgia Cured by Hypodermic Injection of Quinia 419
Malformation of the Heart.................................. 518
Marine Silk.................................................. 263
Marriage between Blood Relations........................ 45
Maryland State Dental Society............................ 573
Massachusetts Dental Society............................ 104
Massachusetts Dental Society. Edward N. Harris, D.D.S.. 133
Medical Professors in Dental Colleges.................. 369
Mental and Manual Labor................................... 629
Mental Labor and Physical Exertion...................... 576
Mixed Chloroform and Ether............................... 315
Morgan's Plastic Gold..................................... 319
Muscle sugar................................................ 630
Muscular Contraction...................................... 574
Mutability of Species...................................... 520
Nævus Maternus............................................ 526
Narceine..................................................... 94
Naso-pharyngeal Polypus, &c.............................. 408
Nature and Treatment of Decay of the Teeth. A Review of Dr. Arthur's Monograph. Prof. H. R. Noel, M.D... 117. 173
Necrosis. New Treatment of................................ 205
Necrosis of Lower Jaw from Phosphorus.................. 96
Necrosis of Upper Jaw from Secondary Syphilis. S. D.. French; D.D.S..... 391
Nervi Nervorum............................................. 630
Nevill, R. P., D.D.S. Fillings of Different Metals in Contact.. 448
Irritation from Vulcanite Plates.... 222
New Anæsthetics............................................ 421
New Appliance. Dr. Henry Clarke's....................... 318
New Base for Artificial Teeth. Colburn's................ 206
New Discovery in Comparative Anatomy.................. 260
New Lingual Muscle........................................ 363
New Microscopic Reagent.................................. 518
New Parasite................................................ 264
New Preparations of Rubber............................... 580
New Process of Preparing Anatomical Specimens......... 417
New Projectile.............................................. 527

New Researches on Cardiac Circulation of Animals....... 365
New Surgical Bandage for Fractures.................... 45
Nitrous Oxide Gas........................ 249. 301. 355. 398. 631
Noel, Prof. Henry R., M.D. Address to Graduating Class
of Baltimore College of Dental Surgery............ 585
Essential Nature of Caries...................... 209. 265. 321
Caries and Necrosis of Bone...................... 425. 482
Osmotic Action.............................. 12. 66
North Carolina Dental Association...................... 52
Obituaries. Dr. John S. Clarke.......................... 52
Dr. A. M. Leslie.......................... 52
John R. McCurdy, Esq...................... 52
Dr. Decatur P. Gregg...................... 372
Dr. Stanley Brown......................... 636
Origin of Petroleum.................................... 51
Osmotic Action. Prof. H. R. Noel, M.D.................. 12. 66
On the Influence of Extreme Cold on Nervous Functions. 224
On the Physiological Action of the Muscles Concerned in the
Movements of the lower Jaw. Dr. T. B. Gunning.... 597
Oxygen in the Market.................................. 522
Ozone.............................................. 316. 516
Peculiarities of Dentition and Dental Disease Hereditary... 103
Physiology of Respiration.............................. 153
Physical History of Various Nations with Special Reference
to their teeth. John Allen, M.D., D.D.S............. 342. 385
Pitting from Small Pox. To Prevent...................... 577
Pivot Teeth............................................ 611
Poisoning of Syphilis and Gonorrhœa..................... 519
Poisonus Hair Washes................................... 627
Practice vs. Theory.................................... 207
Preservation of Anatomical Subjects..................... 418
Preservation of Sulphate of Iron....................... 364
Prevention of Sickness from Chloroform................. 471
Probing Gunshot Wounds................................. 95
Prof. Storer's Lectures................................ 526
Protecting Water from the Action of Lead Pipe............ 630
Pure Silver............................................ 94
Quinine as a Preventative of Malarial Diseases........... 261
Rapidity of Nerve Action................................ 44
Ratio of Deaths from Chloroform......................... 370
Re-Vaccination. Value of Scar, &c...................... 523
Red Blood Corpuscles, Physiological Character and Action of
Prof. R. King Browne, M.D........................ 431. 488
Red Blood Corpuscles. Notes of Observation on Character
of, Prof. R. King Browne, M.D...................... 59
Remarkable............................................ 632
Remarkable Malformation. W. S. Carter, M.D........... 563

Remedy for Cancer.......................................... 583
Reproduction of Bone.......................................... 151
Reproduction of the Crystalline Lens.......................................... 424
Respiratory Changes. Prof. R. King Browne, M.D.......... 330
Restoration of Patients under the Influence of Chloroform.. 517
Resuscitation from Hyperanæsthesia by Chloroform.......... 367
Rise, Progress, and Present Status of Dentistry. H. F.. Bishop, D.D.S.......................................... 347. 379
Root Filling. A. B. Brookins, D.D.S.......................................... 168. 274
Rubber. New Preparations of.......................................... 580
Rubber or Coffer Dam. Barnum's.......................................... 49
Rubber without Sulphur.......................................... 528
Saliva. Andrew S. Cutler, D.D.S.......................................... 537
Salutatory.......................................... 47
Scurvy. Cause of.......................................... 633
Sewing Machines. Effect upon Health.......................................... 51
Simpson's Rubber.......................................... 580
Simpson's Rubber vs., Goodyear Rubber.......................................... 636
Sloughing from Local Anæsthesia.......................................... 263
Spontaneous Cure of Congenital Division of the Palate...... 608
Spontaneous Generation.......................................... 192. 526
Splanchnoscopy.......................................... 472
Styptic Colloid, Richardson's.......................................... 142
Suersen, Dr. Wilhelm Sr. Artificial Palates.......................................... 373
Surgical Clinics of Prof. Edward Warren, M.D.......................................... 424
Surgical Notes.......................................... 624
Surgical Operations.......................................... 416
Susquehanna Dental Association.......................................... 516
Tennessee Dental Association.......................................... 353
Thackston, W. W. H., M.D., D.D.S. The File as a Dental Instrument.......................................... 9
The Algoid Origin of Miasmatic Fevers.......................................... 582
The Blood, Its Progress and Functions. Prof. Rufus King Browne, M.D.......................................... 240
The Convertibility of Electricity and Heat.......................................... 151
The File as a Dental Instrument. W. W. H. Thackston, M.D., D.D.S.......................................... 9
The "Harris Lectures.".......................................... 103. 635
The Persimmon.......................................... 372
The Prudent Live Longest.......................................... 363
Tetrachloride of Carbon as an Anæsthetic. E. Andrews M.D.. 462
Treatment of Pulpless Teeth. Prof. J. Smith Dodge., M.D., D.D.S.......................................... 111
Trichiniasis.......................................... 62. 97
Typographical Error.......................................... *528
Ulcers of the Mouth. Henry S. Chase, D.D.S.......................................... 568
Use and Abuse of Poultices. Dr. Benj. Richardson.......................................... 307

| | | |
|---|---|---|
| Use and Abuse of Tobacco. Sir B. C. Brodie | | 503 |
| Valedictory Address. Prof. Henry R. Noel, M.D | | 585 |
| Valedictory | | 634 |
| Various Risks of Operations. James Paget, F.R.S | 512. | 559 |
| Ventilation | | 413 |
| Virginia Dental Society | 52. | 397 |
| Vulcanite Plates. Plan for Obtaining a more Perfect Fit of, W. Leigh Burton | | 392 |
| Vulcanite Plates, Irritation from. R. P. Nevill, D.D.S | | 448 |
| Vulcanite Rubber for Capping Exposed Nerves | | 476 |
| Warren. Prof. Edward, M.D. Death from Cardiac Neuralgia. | | 21 |
| Weight of the Human Brain | | 524 |
| Wounds by the Chassepot Projectiles | | 521 |

### Dental College Commencements.

### *Baltimore College of Dental Surgery.*

The *twenty-seventh* annual commencement of the Baltimore College of Dental Surgery was held at the Concordia Building, Baltimore, on Thursday evening, February 28, 1867.

The exercises of the occasion were opened with prayer by the Rev. Dr. G. C. M. Roberts. The names of the Graduates were then announced, and the degree of "Doctor of Dental Surgery" was conferred by the Dean of the Faculty, Prof. F. J. S. Gorgas, upon the following gentlemen:—Hugh Wilson Arthur, Md.; Warner Julian Bailey, Miss.; John Robert Barr, Ala.; Thomas Ligget Beckenbaugh, Md.; J. Robson Bromwell, Md.; Walter Bruce, Va.; Andrew Simon Cutler, Ind.; Augustus Boyd Doremus, La.; Joshua Stevenson Dorsey, Md.; John Francis Ruter Dufour, Dist. of C.; Joseph Root England, Md.; John d'Oyley Evans, France; William Farmer, Va.; James Taliaferro Grant, Tenn.; Silon Homer Henkel, Va.; James Hogg, Md.; B. Rush Jennings, Md.; Henry R. Johnson, Va; Harry Galbraith Leas, Pa; Algernon Mosely Lee, M.D., N. C.; Alfred Fitzgerald Malone, Fla.; George William Massamore, Md.; Isaac Carrington Morton, Va.; James William Miller, Va.; Charles A. Norwood, Md.; Robert Lyon Seale, M. D., Ala; Isaiah Simpson, S. C.; Ezekiel Cooper Stockton, M.D., Pa.; George N. Swormstedt, Ind.; Marion Elisha Tarvin, Ala.; John Charles Uhler, Md.

After this part of the ceremony had been concluded, the valedictory address was delivered by Prof. Russell Murdoch. Some remarks were then made by Prof. Thomas E. Bond, at the conclusion of which the benediction was pronounced by the Rev. Dr. Roberts. The large and beautiful hall was filled with the friends and acquaintances of the graduates, and the excellent music of the "Blues' Band" tended greatly to enliven the scene.

It will no doubt be gratifying to the friends of the Baltimore College to announce that the number of matriculants was almost double that of the previous session, and that the graduating class numbered *thirty-one.*

The faculty were much gratified at the presence of a number of the former graduates of the institution, gentlemen whom they feel proud to number among its Alumni.

The following change in the faculty was also announced; Dr. H. H. Keech having resigned the Demonstratorship of Operative Dentistry, Dr. Hugh McGinnis Grant, of Abingdon, Virginia, of the class of 1855, has been appointed Demonstrator of Operative Dentistry.

---

### *Ohio College of Dental Surgery.*

The *twenty-first* annual commencement of this College was held in the hall of the College Building, Cincinnati, March 6th, 1867. The degree of "Doctor of Dental Surgery" was conferred by Dr. James Taylor, President of the Board of Trustees, on the following gentlemen:

W. C. Stanley, F. McGinniss, F. Peabody, G. W. Field, H. L. Ambler, J. T. Child, B. Eaton, P. T. Clark, W. A. Grahame, R. F. Ludwig, J. R. Irelan, J. Ropp.

The valedictory was read by Prof. Spalding, on account of the absence of Dr. Richardson, by whom it was written.

The graduating class numbered twelve.

---

### *Pennsylvania College of Dental Surgery.*

The *eleventh* annual commencement of this College was held at Musical Fund Hall, Philadelphia, March 1st, 1867.

The degree of "Doctor of Dental Surgery" was conferred by Dr. W. W. Fouche, upon the following gentlemen, who were regular students in attendance upon the lectures, twenty-six in number:

Stephen Armos, Cuba; John Aspinwall, Jr., Mass.; Edward M. Beesly, N. J.; Charles Bulkley, Pa.; John N. Crouse, Ill.; Charles H. Darby, Mo.; Frank Darby, N. Y.; Squire C. Dayan, N. Y.; James W Gurley, Or.; Robert Huey, Pa.; James Lewis, Vt.; David R. Martin, Pa.; Mariano Martorell, Porto Rico; John Q. McDavid, S. C.; Henry W. Moore, Pa.; Gonzalo Orue, Cuba; Casimiro Portillo, Cuba; George L. Rauch, Pa.; John S. Smith, Pa.; James A. Sheldon, N. Y.; Clinton W. Strang, N. Y.; James Taylor, England; George R. Thomas, Pa.; Francisco Vega, Porto Rico; H. Meredith White, M.D., Pa.; Joseph F. Winslow, N. Y.

The same degree was also conferred upon the following gentlemen, who have been in practice since 1852, but who were not required to attend the lectures of the institution, a course which it will be seen by reference to the proceedings of the Association of Dental Colleges, is disapproof by all the other Colleges:

G. C. Brown, N. J.; J. F. Leaming, N. J.; D. R. Greenlee, Pa.; J. H. Githens, Pa.; Spenser Roberts, Pa.; Amos Wirt, Pa.; A. R. Robbins, Pa.; Benjamin Wood, N. Y.; C. A. Marvin, N. Y.; W. C. Parks, N. Y.; G. H. Perine, N. Y.; C. E. Francis, N. Y.; W. B. Hurd, N. Y.; T. Burgh, N. Y.; S. Hassell, N. Y.; A. L. Northrop, N. Y.; Enos G. Ray, N. Y.; T. H. Musgrove, Md.; W. W. Russell, Mass.; J. A. Salmon, Mass.; E. G. Leach, Mass.; D. S. Dickerman, Mass.; Chester Heath, N. H.

The valedictory was delivered by Prof. T. L. Buckingham.

---

### *Philadelphia Dental College.*

The *fourth* annual Commencement of this College was held at Musical Fund Hall, Philadelphia, March 1, 1867.

The degree of "Doctor of Dental Surgery" was conferred by Rev. Richard Newton, D.D, upon the following gentlemen, thirty in number:

Julian J. Anderson, Mass.; Stephen T. Beale, Jr., Penn.; James E. Blanchard, La.; Frederick K. Crosby, Conn.; Charles M. Curtis, Penn.; Roger Cutlar, N. C.; Charles V. Du Bouchett, Penn.; George P. Franklin, Penn.; Henry L. Gilmour, Ireland; Edward Goertz, Germany; Daniel G. Harkins, Mass.; William C. Head, Penn.; Arthur Holbrook, Wis.; William H. Howard, Penn.; Frank A. Hunter, N. Y.; John G. James, N. C.; M. Lukens Long, Penn.; Andrew F. McAvenney, N. B.; Lewis P. Meredith, Ohio; Edward D. Moore, Ohio; George B. Morris, W. Va.; George S. Nyce, Penn.; John J. Pitts, N. Y.; John Powers, Maine; Henry A. Robinson, Maine; David D. Smith, Mass.; Leopold M. Townsley, Mo.; Carl R. Walther, Germany; Marshall H. Webb, Penn.; Otis C. White, Mass.

The valedictory was delivered by Prof. Thomas Wardle.

---

### *New York College of Dentistry.*

The *first* annual Commencement of this College was held at Steinway Hall, New York, March 6th, 1867. The degree of "Doctor of Dental Surgery" was conferred by Prof. Eleazar Parmly upon the following gentlemen, nine in number:

C. D. Allen, W. C. Horne, J. W. Lyon, N. Y.; J. F. P. Hodgson, Ithaca, N. Y.; R. W. Browne, Conn.; G. Bernard, Wash. Ter.; W. D. Tucker, Tenn; W. Dutch, California; C. F. Meyer, Germany.

The valedictory was delivered by Dr. W. W. Allport.

### BOOK NOTICES.

*Nature and Treatment of Decay of Teeth.*—By R. Arthur, M. D., D. D. S.—Baltimore, John Murphy & Co., 1867. A monograph plain and practical, addressed to the public as well as to the profession, and therefore written in a style suited to the popular taste. It is the duty of the educated practitioner to instruct his patients, and without such a determination to instruct, he is unfaithful to his dignity as a member of a liberal profession. Our patients look for such service at the hands of educated men, and they are willing to receive it, if it be rendered with a generous sympathy and a courteous respect. Dr. Arthur has taken one of the various methods by which, in this age of advancement, we may impart knowledge. We make no comments upon its contents, as it will be thoroughly reviewed by Prof. H. R. Noel, in an article for our June number.

Messrs. Murphy & Co, have gotten the book up in admirable style. It is printed on good paper with clear type, and its wood cuts with few exceptions are excellent

*Transactions of the American Dental Association.*—Dr. L. D. Shepard, of Salem, Mass., Chairman of the Publishing Committee, announces that this work will be issued some time during the present month. As it will contain the proceedings of the American Dental Convention from the first meeting to that of July, 1866, the work will be an interesting one to the dental profession.

*Harris' Medical and Dental Dictionary,* (*new edition,*) carefully revised and enlarged by Prof. F. J. S. Gorgas.—This work is now in the hands of the printer, and will be issued in a few months. Publishers, Lindsay & Blakiston, Philadelphia.

### EDITORIAL DEPARTMENT.

*A Word of Explanation.*—Our readers will notice the absence of Selected Articles in this number. We had several interesting papers marked for our selected department, but they were crowded out by the quantity of original articles kindly placed at our disposal by our friends. We also apologize to those of our contributors whose papers have been left out for want of room. They shall appear at the earliest possible date.

